# The Impact of Experience Versus Decision Aids on Patient Preference Toward Virtual Care

**DOI:** 10.1089/tmr.2024.0001

**Published:** 2024-03-21

**Authors:** Aaron R. Prater, Jack T. McConnell, Nikhil R. Yedulla, Edward L. Peterson, Trevor R. Banka, Charles S. Day

**Affiliations:** ^1^ Department of Orthopedic Surgery, Henry Ford Health, Detroit, Michigan, USA.; ^2^ Wayne State University School of Medicine, Detroit, Michigan, USA.

**Keywords:** decision aid, education, telehealth, telemedicine

## Abstract

**Introduction:**

Virtual care utilization has increased in recent years bringing questions of how to best inform patients regarding their use. Decision aids (DAs) are tools created to assist patients in making informed decisions about their health care. This study seeks to determine whether a DA or previous experience could better educate and influence patient's preference on virtual care.

**Methods:**

One hundred fifty participants from an orthopedic clinic of a multi-hospital system were divided into three groups. Group 1 (Virtual Care Cohort) had at least one previous virtual care visit and was surveyed with the Telemedicine Satisfaction Questionnaire (TSQ). Group 2 (In-person with Decision Aid) and Group 3 (In-person without Decision Aid) had no virtual care experience. Group 2 received a validated virtual care DA with a knowledge test. Both groups were also administered the TSQ.

**Results:**

After the DA, patients improved their score on 3 of 4 virtual care knowledge questions. Each cohort demonstrated a positive perception of virtual care; however, the specific reasons for their favorable views varied. The DA cohort did not show increased preference toward virtual care compared with the non-DA group and only responded significantly higher regarding encounter comfort. Patients with previous experience in virtual care responded most favorably to the majority of survey questions regarding their virtual care preferences when compared with both virtual care naive cohorts.

**Discussion and Conclusion:**

We found that patient experience was the most important factor in influencing patient preference toward virtual care. Although the DA increased their virtual care knowledge it did not increase their preference; therefore, efforts should be placed at encouraging patient to experience virtual care.

## Introduction

Virtual care utilization has significantly increased since the onset of the COVID-19 pandemic, with virtual visits accounting for 6.5% of all clinic visits in a 2020 study of >50,000 providers.^1^ This growth has offered a new method for patient–physician communication that overcomes geographical and scheduling barriers, reduces patient wait time, increases patient compliance, and improves communication across multiple treatment centers.^2–6^ However, concerns regarding the lack of defined policies within hospitals, the inability to perform a physical examination, and potential discrepancies in health care access have been raised.^7–9^ Therefore, understanding patient perspectives of virtual care is crucial in assessing its role in medical settings.^10,11^

Previous literature in specialties such as surgery and obstetrics has shown that patients have positive views of virtual care due to decreased travel times, increased convenience and reliability, and decreased cost.^12–16^ In addition, studies in the orthopedic fields of sports medicine and hand found 88% and 100% of patients, respectively, were either “satisfied” or “very satisfied” with their virtual appointment.^17,18^

However, older patients, shorter visit duration, and concerns about misdiagnosis influence patient hesitancy toward telehealth.^19,20^ Moreover, patients have also expressed reluctance toward virtual care due to technological challenges and the inability to be physically examined.^13^ Although virtual care has been used for all visit types, studies have shown that orthopedic providers have positive views of virtual care for return or postoperative visits and negative views for new patient appointments.^21^

The increasing evidence that patients want a more active role in their treatment plans has led to the development of the shared decision-making model, which considers a patient's past experiences and values.^22–25^ In addition, the implementation of patient decision aids (DAs) have been incorporated into the care model to help patients choose management and treatment options that align with their values.^26–29^ It is essential to note that the shared decision-making model acknowledges that there are usually multiple ways to address or treat a patient's condition.^30^

Thus, the purpose of the DAs is to increase patient knowledge, reduce decisional conflict, and enhance patient involvement in treatment decisions.^31–33^ Although one study in orthopedics has validated the use of a DA for patients considering a virtual care appointment, there are no published studies in orthopedic surgery that specifically compares patient preference toward virtual care with and without the use of a DA.^34^

The purpose of our study aims to assess patient preference toward virtual care utilizing a validated DA in patients who have and have not experienced a virtual visit. First, we hypothesize that administering a virtual care DA will improve patients' knowledge of virtual visits. Second, we hypothesize that patients will exhibit an increased preference toward virtual visits after being administered a virtual care DA.

## Methods

This study was conducted in a multi-hospital academic and community care health system involving 5 hospitals and 15 clinics. Out of an 85-member service line, the surgeon with the greatest number of virtual visits was chosen for the study to minimize the confounding variables caused by intra-provider variability. A power analysis was performed and determined that 150 patients were adequate to provide statistical significance in reported patient preference between patients who did and did not receive a DA.

A previously validated virtual care satisfaction survey, the Telemedicine Satisfaction Questionnaire (TSQ),^35^ and previously validated virtual care DA^34^ (Supplementary Fig. S1) were utilized for this study. A secondary 4-question survey (knowledge test) was used for patients who received the DA to evaluate participant's understanding. This study received institutional review board approval.

### Data collection

**FIG. 1.**
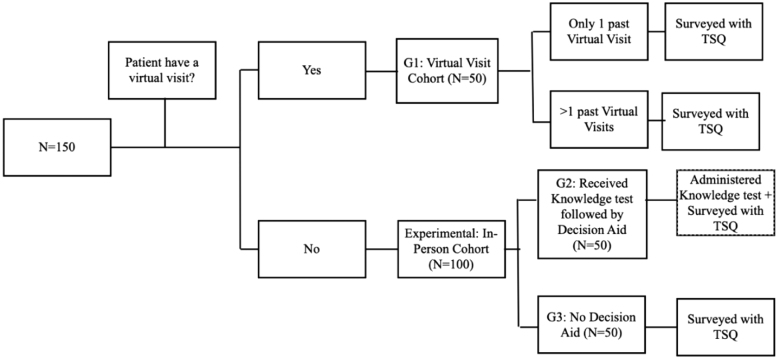
Flowchart displaying the division of patients into their respective cohorts. All steps of the methodology completed by each group are included. TSQ, Telemedicine Satisfaction Questionnaire.

Data collection took place from March 2021 to February 2022. Patients aged >18 years were selected for the study and stratified into three equal cohorts (Fig. 1). Participation in this study was optional, and patients received no compensation for doing so; completion of a validated 6-item Orientation-Memory-Concentration cognitive assessment was required before enrollment and was administered by the research assistant.^36^ All surveys were completed using RedCap software and administered by a research assistant in the clinic using a tablet or virtually using a link sent through RedCap to the patient's e-mail.

Three cohorts made up this study. Group 1 patients (Virtual Care Cohort) were informed of the study during their virtual visit with the orthopedic provider. They were subsequently e-mailed the TSQ the same day if agreeable to project participation. A reminder e-mail was sent 1 week after the initial contact with two additional reminders in 1-week intervals. If the patient did not respond or they completed the survey, no further contact was pursued. Only one submission per survey was collected. Group 2 (In-person with Decision Aid) patients were individuals seen in the clinic who had not previously experienced a virtual visit with any provider.

Participants were given a pre-DA knowledge test to determine their understanding of telehealth visits. Subsequently, the DA was given to the patient, and they were allowed a self-determined period to read the DA. Participants were then readministered the knowledge test to record understanding and then asked to complete the TSQ survey. Group 3 (In-person without Decision Aid) patients were individuals seen in the clinic who also had not previously experienced a virtual visit. They were administered the TSQ survey without the DA. Participants were randomized to Group 2 (*n* = 50) and 3 (*n* = 50) using a blinded research member and the patient's medical record number.

### Data analysis

We analyzed each of the four knowledge test questions individually and the aggregate score before and after receiving the DA. Each of the three cohorts were analyzed simultaneously with a Kruskal–Wallis test and then individually with three pairwise comparisons using two-sample Wilcoxon tests. The knowledge test question results were analyzed using a matched-pairs signed-rank Wilcoxon test. When multiple comparisons were examined, the results were adjusted using Hochberg's methods. The questions targeted common virtual care misconceptions, uses for, and equipment needed for telehealth visits.

Each of the 10 questions in the TSQ were utilized in this study and were scored on a 1–5 Likert scale (1 = Strongly Agree, 2 = Agree, 3 = Neutral, 4 = Disagree, and 5 = Strongly Disagree). Individual questions were directly compared between the three groups. A multivariate analysis was performed to determine the significance of the responses between the three groups. Group 1 had a subgroup analysis, examining single virtual visits (*n* = 23) versus multiple virtual visits (*n* = 27). The TSQ results between these two subgroups were then directly analyzed against each other. A *p*-value of <0.05 was considered statistically significant.

## Results

This study enrolled 150 participants and placed them into three equal-sized cohorts (*n* = 50) (Fig. 1). 50/68 patients queried for Group 1 responded to the survey (73.5% response rate, *n* = 50). All 100 patients approached in the clinic for Groups 2 and 3 participated in the study. The age distribution between the three cohorts was equivalent and insignificant (*p* = 0.523). The average age in the virtual visit cohort was 62.6 ± 10.9, 64.3 ± 11.8 in the DA cohort, and 65.4 ± 9.2 in the no-DA cohort (Table 1). Sex, race, education, and employment were equally distributed among the three groups (Table 1).

**Table 1.** A Comparison of Demographic Information (Age, Sex, Race, Highest Level of Education, and Employment Status) of the Patients in the Three Cohorts

Patient demographics	Virtual visit (G1)	DA (G2)	No DA (G3)	***p***-Value			
				Overall	1 v 2	1 v 3	2 v 3
Age (years)	65.4 ± 9.2	62.6 ± 10.9	64.3 ± 11.8	0.523	0.249	0.622	0.544
Sex				0.461	0.288	1.000	0.288
Male	28%	38%	28%				
Female	72%	62%	72%				
Race				0.285	0.184	0.500	0.17
White	66%	50%	54%				
Black	28%	44%	38%				
American Indian	—	2%	—				
Asian	2%	4%	—				
Other	4%	—	6%				
Education				0.079	0.062	0.130	0.617
<High school	—	—	2%				
High school	36%	34%	44%				
Trade school	6%	12%	6%				
Associates degree	14%	28%	28%				
Graduate school	28%	24%	16%				
Postgraduate school	14%	—	2%				
Other	2%	2%	2%				
Employment				0.073	0.054	0.078	0.589
Full time	24%	40%	48%				
None, not looking	—	6%	2%				
None, looking	4%	4%	4%				
Retired	56%	48%	40%				
Work from home	12%	2%	2%				
N/A	4%	—	4%				

*N* = 50 for each cohort.

### Knowledge test

**FIG. 2.**
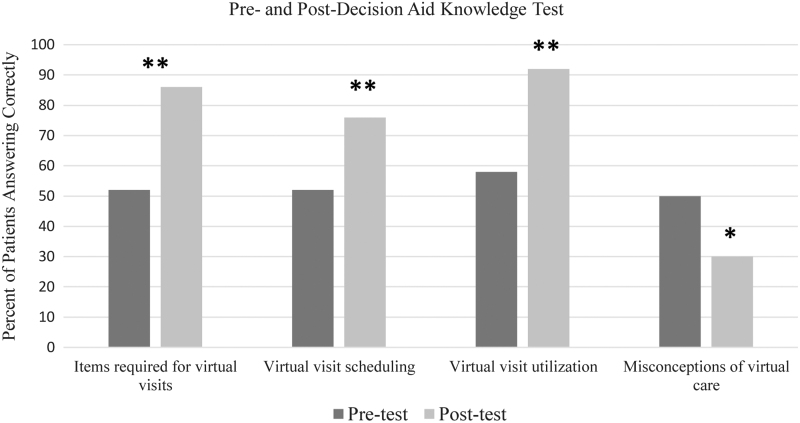
Comparison of the pre- and post-DA results for the DA experimental cohort. *p < 0.05; **p < 0.01. DA, decision aid.

When comparing participants' responses in the DA group who completed the pre- and post-DA knowledge assessment, improvement was seen in three of the four questions (Fig. 2). When responding to what was required for a virtual visit (Q1), 52% of participants answered correctly, whereas 86% answered correctly after reading the DA (*p* = 0.001). Regarding scheduling (Q2), 52% of participants answered correctly before the DA and 76% after (*p* = 0.007). When asked about the utilization of virtual care (Q3), 58% responded correctly before receiving the DA and 92% after (*p* = 0.001). Finally, when asked about common misconceptions of virtual care (Q4), 50% of participants answered correctly before, and 30% responded correctly after reading the DA (*p* = 0.018).

### TSQ responses: Groups 2 v 3

**FIG. 3.**
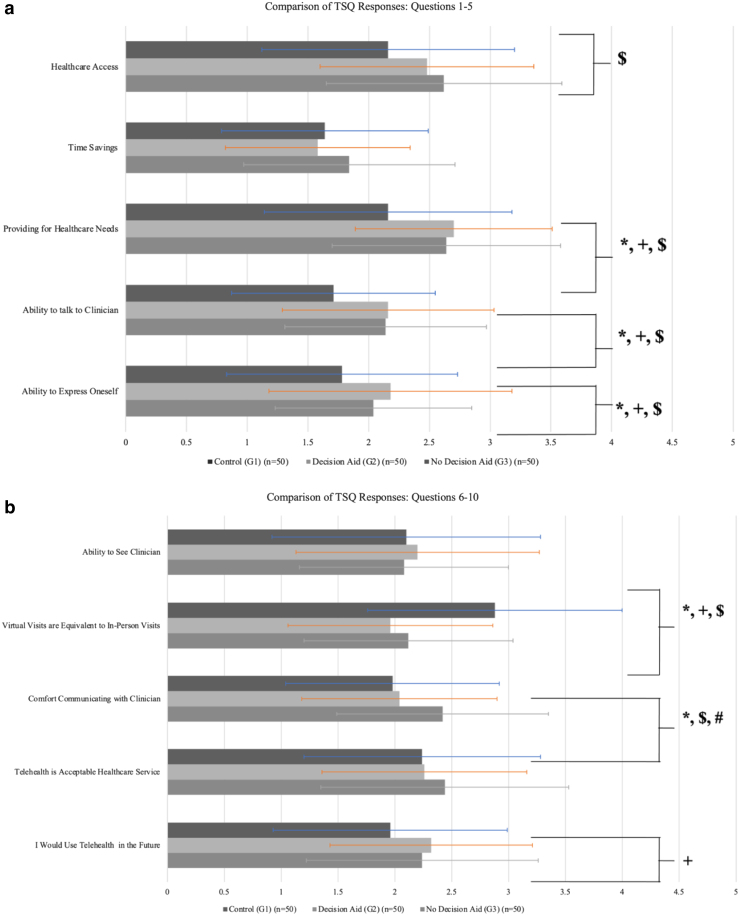
Comparison of the TSQ responses between the three cohorts (**a** [*top*] = Questions 1–5; **b** [*bottom*] = Questions 6–10). *Represents *p* < 0.05 between all three cohorts. +Represents *p* < 0.05 between the Control (Group 1) and DA (Group 2) groups. $Represents *p* < 0.05 between the control (Group 1) and non-DA (Group 3) groups. #Represents *p* < 0.05 between the DA (Group 2) and non-DA (Group 3) groups. *N* = 50 for each cohort.

When comparing the responses of the two in-person cohorts that did and did not receive the DA, only one question resulted in a significant difference. When asked if patients would feel comfortable having a medical visit using telehealth services, the DA group had a mean response of 2.04 ± 0.86 versus 2.42 ± 0.93 in the non-DA group (*p* = 0.036) (Fig. 3a, b and Table 2). No other question elicited a significant response between these two cohorts.

**Table 2.** Comparison of the Telemedicine Satisfaction Questionnaire Responses Between the Three Cohorts

TSQ results	Control (G1) (***n*** = 50)	DA (G2) (***n*** = 50)	No DA (G3) (***n*** = 50)	***p***-Value			
				Overall	1 v 2	1 v 3	2 v 3
Health care access (Q1)	2.16 ± 1.04	2.48 ± 0.88	2.62 ± 0.97	0.053	0.083	0.023^*^	0.418
Time savings (Q2)	1.64 ± 0.85	1.58 ± 0.76	1.84 ± 0.87	0.201	0.790	0.167	0.095
Providing for health care needs (Q3)	2.16 ± 1.02	2.70 ± 0.81	2.64 ± 0.94	0.007^*^	0.003^*^	0.014	0.718
Ability to talk to clinician (Q4)	1.71 ± 0.84	2.16 ± 0.87	2.14 ± 0.83	0.003^*^	0.004^*^	0.004^*^	0.901
Ability to express oneself (Q5)	1.78 ± 0.95	2.18 ± 1.00	2.04 ± 0.81	0.037^*^	0.026^*^	0.032^*^	0.554
Ability to see clinician (Q6)	2.10 ± 1.18	2.20 ± 1.07	2.08 ± 0.92	0.755	0.473	0.652	0.732
Virtual visits are equivalent to in-person visits (Q7)	2.88 ± 1.12	1.96 ± 0.90	2.12 ± 0.92	0.001^*^	0.001^*^	0.001^*^	0.372
Comfort communicating with clinician (Q8)	1.98 ± 0.94	2.04 ± 0.86	2.42 ± 0.93	0.027^*^	0.590	0.014^*^	0.036^*^
Telehealth is acceptable health care service (Q9)	2.24 ± 1.04	2.26 ± 0.90	2.44 ± 1.09	0.691	0.861	0.421	0.511
I would use telehealth in the future (Q10)	1.96 ± 1.03	2.32 ± 0.89	2.24 ± 1.02	0.056	0.022^*^	0.112	0.403

^*^ *p* < 0.05. *N* = 50 for each cohort.

TSQ, Telemedicine Satisfaction Questionnaire.

### TSQ responses: Groups 1 v 2

More differences were appreciated when comparing the virtual care group to those that received the DA. Five of the 11 questions resulted in a significant difference in preference toward virtual care, although only 1 question was more significant in the DA cohort. When asked if virtual visits were the same as in-person, the mean response from the DA cohort was 1.96 ± 0.90 versus 2.88 ± 1.12 in the virtual visit cohort (*p* = 0.001) (Fig. 3a, b and Table 2).

The other four questions elicited more favorable responses in the virtual visit cohort. When asked if virtual care would provide their health care needs (*p* = 0.003), if they felt they would be able to hear their provider adequately (*p* = 0.004), if they could express their medical concerns (*p* = 0.026), and if they would use telehealth in the future (*p* = 0.026), responses showed a higher preference toward virtual care among the virtual care cohort (Fig. 3a, b and Table 2).

### TSQ responses: Groups 1 v 3

When comparing the responses from the group that did not receive the DA to the virtual group, 6 of the 10 questions had significant results. Similarly, when asked if virtual visits were the same as in-person visits, the non-DA group showed a greater preference for virtual visits with a mean response of 2.12 ± 0.92 versus 2.88 ± 1.12 (*p* = 0.001) (Fig. 3a, b and Table 2).

In addition, in response to if virtual care would increase access to health care (*p* = 0.023), if virtual care would provide for their needs (*p* = 0.014), if they would be able to hear their provider adequately (*p* = 0.014), if they could express their medical concerns (*p* = 0.032), and if they would feel comfortable using telehealth (*p* = 0.014), the virtual care group answered the survey more favorably (Fig. 3a, b and Table 2).

### TSQ responses: All groups

Three survey questions did not provide a significant difference among the cohorts. All three cohorts answered similarly to if telehealth would save them time when receiving health care. The DA group had a mean response of 1.58 ± 0.76, the non-DA group 1.84 ± 0.87 (*p* = 0.095), and the virtual care group 1.64 ± 0.85 (*p* = 0.790) (Table 2). In response to if participants felt they would be able to see their provider using telehealth (*p* = 0.755) and if telehealth was an acceptable modality for receiving health care (*p* = 0.691), no significance was appreciated (Fig. 3a, b and Table 2).

### TSQ responses: Group 1A v 1B

The virtual visit group was further stratified based on whether they had experienced one virtual visit (*n* = 23) or if they had experienced multiple virtual visits (*n* = 27) (Supplementary Fig. S2). Each of the 10 TSQ survey questions were analyzed and failed to show any significant difference in the responses between the two groups.

## Discussion

As virtual care becomes increasingly utilized in medicine, it is essential to understand how patients perceive this technique and if educational tools, such as DAs, can educate patients and alter decision-making. Our study found that utilizing a DA effectively improves patients' understanding of virtual care, as scores in 3 of the 4 knowledge test questions improved after administration of the DA.

Despite increased knowledge, TSQ survey results showed that all three cohorts in the study had a favorable view of virtual care, with no clear trends identified that demonstrated the DA increased patient preference toward virtual visits. Patients who had a virtual visit were shown to have increased preference toward virtual care, suggesting that experience, not a DA, is ultimately the most significant tool for altering a patient's preference in the modality in which they receive care.

In a study out of Michigan, Yedulla et al.^21^ developed a virtual care DA and administered it to patients with a knowledge test before and after reading the DA. This study enrolled 124 patients and sought to determine if their DA could increase their knowledge of virtual care and help them decide which care modality was suitable for them. After administering the DA, the authors found a 15.3% increase in knowledge, with 79.8% of patients feeling that they fully understood their care modality options.

However, this study did not use a previously validated telehealth survey, such as the TSQ, to gauge patients' preferences for virtual care and whether they changed after administering a DA. In addition, the patients surveyed were not compared with those who had already experienced telehealth visits with a health care provider. A systematic review/meta-analysis by Chaudhry et al.^37^ analyzed patient and surgeon satisfaction with telemedicine in orthopedic care and patient-reported outcomes in 12 American and German health systems.

The study found no appreciable difference in preference between in-person and virtual visits. Although validated telehealth surveys were used in these studies, they did not compare patient preferences of patients that had not experienced a virtual visit to those that had. In addition, none of the studies in this review utilized a DA or studied its impact on patient decisions. Our study used a DA and a validated telemedicine satisfaction survey to directly compare patients' preferences that had and had not received telehealth and whether these preferences could be influenced through a DA.

Our study has certain limitations that need to be acknowledged. First, the study was performed at a single tertiary orthopedics joint clinic with patients from a single provider. Enrolling patients from a single provider allowed us to limit confounding variables related to intra-physician technique and preference for provider style, although possibly introducing bias in our sample population studied. Moreover, our patient recruitment satisfied the needs of our power analysis.

Another limitation was that in-person patients were enrolled in the study after the completion of their office visit. This could have introduced a selection bias as patients who did not wish to participate could have left before a research assistant attempted to enroll them. This practice was necessary to preserve the workflow and minimize disruption in a busy clinic. However, because the patients recorded were then randomized, we feel that this did not introduce another confounding variable once patients were assigned to groups. These limitations need to be considered when interpreting the results of the study.

## Conclusion

In conclusion, our study demonstrated that patient experience is the most significant factor affecting their preference for telehealth. Our study found increased patient knowledge regarding virtual care after administering a virtual care DA; however, there was no difference in their overall preference for the care modality. In addition, our findings support that virtual care patients had an increased comfort level compared with traditional visits.

Although the exact reasons are unknown, patients felt more comfortable expressing themselves when not face-to-face with a physician. Further studies are needed to expand to more clinics and clinicians to determine if these findings are consistent with the broader population. Based on our research, health care systems should encourage patients to experience a virtual visit as the most effective way of utilizing this care modality.

## Supplementary Material

Supplemental data

Supplemental data

## Abbreviations Used

DA: decision aid
TSQ: Telemedicine Satisfaction Questionnaire
