# Supplementary material for: The Impact of Experience Versus Decision Aids on Patient Preference Toward Virtual Care
Source: Telemed Rep. 2024 Mar 21;5(1):59–66. doi: 10.1089/tmr.2024.0001 (PMC10979688; doi:10.1089/tmr.2024.0001)
Supplement: Supplemental data [file Suppl_FigureS1.pdf]

Patient Decision Aid for Individuals Considering Video Visits vs. Traditional Office Visits

What this Patient Decision Aid is for:
This Patient Decision Aid is intended to help patients and health professionals in discussion among preference for Video Visits (VV) to Traditional Office Visits (TOV) for hand follow-up.

What is a Video Visit?
A Video Visit is a personal video chat communication between a health professional and patient using an electronic device via a secure application providing clinical services without the need for a physical in-person visit.

What Matters to You?
Your personal feelings are just as important as the medical facts. Think about what matters to you in this decision and show how you feel about the following statements.

	Video Visit	Traditional Office Visit
What is usually involved?	• Patients communicate with physician via video call on a personal device at a pre-determined time<br>• Takes place as a follow-up appointment to surgeries or general consultations<br>• No medical diagnostics or devices are required at the patient location<br>• Can prescribe medications and provide a script for splints, imaging, and Physical Therapy.<br>• Can see discoloration, swelling, and asymmetry.	• Patients physically arrive at clinic to meet with physician at a pre-determined time<br>• Patients need to come in to the office for a follow-up appointment to surgeries or general consultations<br>• Patient is screened and is required to fill out paperwork upon arrival.
What are the differences?	• Unable to perform thorough and accurate physical examination of patient<br>• Unable to administer in-clinic procedures (i.e. injections)<br>• Involve primarily the physician	• Able to perform thorough and accurate physical examinations<br>• Able to provide procedures (i.e. cortisone injections)<br>• Involve the entire care team (i.e. RN, medical assistant)
What is the transportation and travel time requirements?	• No transportation or travel time to clinic necessary<br>• Visit can take place at home, work, or any other remote location	• Transportation and travel time to clinic required<br>• Potential modes include walking, driving, public transportation, and ride sharing
What are the technological requirements?	• Laptop, desktop with a camera, phone, tablet, or similar device with network connectivity (WIFI or Cellular)<br>• Active secure patient portal account and access to portal via mobile app/web	• None required
What is the cost?	• Same	• Same
