# Supplementary material for: The Impact of Experience Versus Decision Aids on Patient Preference Toward Virtual Care
Source: Telemed Rep. 2024 Mar 21;5(1):59–66. doi: 10.1089/tmr.2024.0001 (PMC10979688; doi:10.1089/tmr.2024.0001)
Supplement: Supplemental data [file Suppl_FigureS2.pdf]

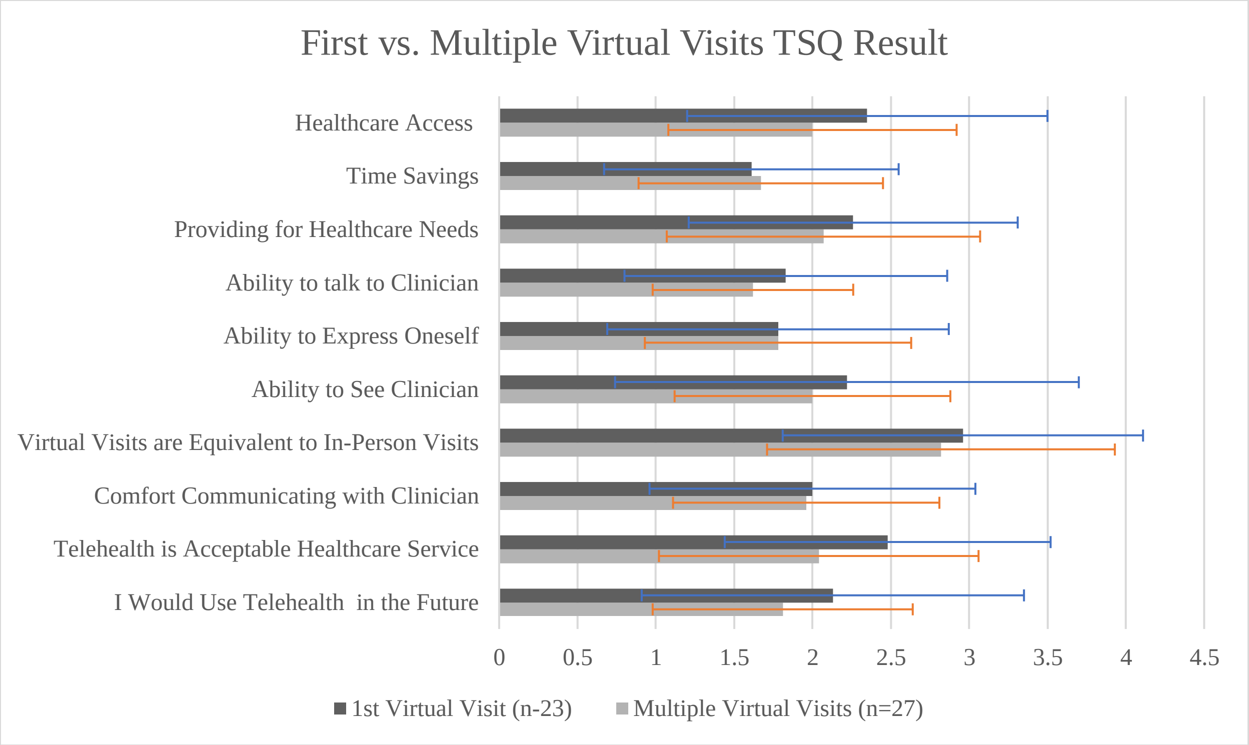

Supplemental Figure 2. Comparison of the TSQ results between first time and multiple time virtual visit patients within the control cohort. ***** represents $p < 0.05$. N=23 for first time virtual visits and N=27 for multiple virtual visits.
